# PDGF Suppresses the Sulfation of CD44v and Potentiates CD44v-Mediated Binding of Colon Carcinoma Cells to Fibrin under Flow

**DOI:** 10.1371/journal.pone.0041472

**Published:** 2012-10-08

**Authors:** Christina S. Alves, Konstantinos Konstantopoulos

**Affiliations:** 1 Department of Chemical and Biomolecular Engineering, The Johns Hopkins University, Baltimore, Maryland, United States of America; 2 Johns Hopkins Physical Sciences, Oncology Center, The Johns Hopkins University, Baltimore, Maryland, United States of America; 3 Institute for NanoBioTechnology, The Johns Hopkins University, Baltimore, Maryland, United States of America; Ottawa Hospital Research Institute, Canada

## Abstract

Fibrin(ogen) mediates sustained tumor cell adhesion and survival in the pulmonary vasculature, thereby facilitating the metastatic dissemination of tumor cells. CD44 is the major functional fibrin receptor on colon carcinoma cells. Growth factors, such as platelet-derived growth factor (PDGF), induce post-translational protein modifications, which modulate ligand binding activity. In view of the roles of PDGF, fibrin(ogen) and CD44 in cancer metastasis, we aimed to delineate the effect of PDGF on CD44-fibrin recognition. By immunoprecipitating CD44 from PDGF-treated and untreated LS174T colon carcinoma cells, which express primarily CD44v, we demonstrate that PDGF enhances the adhesion of CD44v-coated beads to immobilized fibrin. Enzymatic inhibition studies coupled with flow-based adhesion assays and autoradiography reveal that PDGF augments the binding of CD44v to fibrin by significantly attenuating the extent of CD44 sulfation primarily on chondroitin and dermatan sulfate chains. Surface plasmon resonance assays confirm that PDGF enhances the affinity of CD44v-fibrin binding by markedly reducing its dissociation rate while modestly increasing the association rate. PDGF mildly reduces the affinity of CD44v-hyaluronan binding without affecting selectin-CD44v recognition. The latter is attributed to the fact that CD44v binds to selectins via sialofucosylated O-linked residues independent of heparan, dermatan and chondroitin sulfates. Interestingly, PDGF moderately reduces the sulfation of CD44s and CD44s-fibrin recognition. Collectively, these data offer a novel perspective into the mechanism by which PGDF regulates CD44-dependent binding of metastatic colon carcinoma cells to fibrin(ogen).

## Introduction

Fibrinogen is a 340-kDa glycoprotein composed of two identical disulfide-linked subunits, each of which is formed by three distinct polypeptide chains, Aα, Bβ, and γ [1]. Although fibrinogen is relatively inert in the circulation, upon conversion to fibrin it interacts with a variety of proteins and cells to participate in numerous (patho)physiological processes. Fibrinogen plays a key role in blood clotting cascade whereby thrombin-mediated cleavage of the two N-terminal fibrinopeptides A and B (residues Aα(1–16) and Bß(1–14), respectively) from the central region of fibrinogen results in fibrin formation [1]. It is now widely accepted that fibrin(ogen) is involved in the hematogenous dissemination of tumor cells, including colon carcinomas. The contribution of fibrin(ogen) to metastasis has been established by the use of fibrinogen-deficient mice, which exhibit a profound inhibition of experimental and spontaneous metastasis relative to wild-type control mice [2]; [3]; [4]; [5]. It is believed that platelet-fibrin(ogen) clots surrounding tumor cells protect them from immunological and physiological stresses in the bloodstream and facilitate their lodging to the pulmonary vasculature [6]. This hypothesis is corroborated by in vivo data, which reveal that in mice lacking functional natural killer (NK) cells, fibrin(ogen) deficiency was no longer a significant determinant of metastatic potential [3].

We have recently reported that CD44 is the major functional fibrin receptor on colon carcinoma cells [7], [8]. CD44 is a type I transmembrane glycoprotein encoded by a single gene and has at least 20 exons [9]. Exons 1–5, 16–18 and 20 are spliced together to form the smallest *CD44* transcript known as standard form (CD44s) [10] with an estimated molecular mass by SDS-PAGE of 80–95 kDa. At least ten exons (6–15; typically identified as v1–v10) can be alternatively spliced and inserted at a single site within the membrane proximal portion of the extracellular domain to give rise to multiple variant isoforms of CD44 (CD44v) with a molecular mass up to 250 kDa [9], [10]. CD44s can be found in most tissues of the adult organism, whereas the larger variant isoforms are expressed in only a few epithelial tissues, mainly in proliferating cells, and in several cancers [9].

CD44 undergoes extensive post-translational modifications resulting from the attachment of carbohydrates to *N*- and *O*-linked glycosylation sites of the extracellular domain, and of glycosaminoglycans (GAGs) such as chondroitin sulfate, dermatan sulfate [11], heparan sulfate [12], and keratan sulfate [13]. *N*- and *O*-glycosylation as well as GAG modifications of CD44v or CD44s may negatively or positively influence ligand binding [14]. We recently reported that CD44s-fibrin recognition has an absolute requirement for *N-*, but not *O-*, linked glycans [8]. This scenario is completely reversed for CD44v-fibrin binding [8]. Moreover, the presence of chondroitin and dermatan sulfates on CD44 standard and variant isoforms facilitates fibrin recognition [8]. Sulfation of proteins, lipids, polysaccharides and/or GAGs can also modulate ligand binding activity, including CD44v-fibrin recognition [8].

A variety of growth factors and cytokines have been reported to influence the post-translational modifications such as glycanation and sulfation of glycoproteins. For instance, tumor necrosis factor-α (TNF-α) increases the carbohydrate sulfation of CD44 in the SR91 myeloid cell line [15]. Incubation of resting fibroblasts with platelet-derived growth factor (PDGF) induced glycanation of CD44s with chondroitin and dermatan, but not heparan, sulfates, and potentiated migration into fibronectin/fibrin gels [11].

Given the important role of PDGF in modulating tumor cell function [16] and its ability to regulate post-translational protein modifications [11], we aimed to delineate its effect on CD44v- versus CD44s- fibrin recognition pertinent to the process of cancer metastasis.

## Results

### PDGF increases the adhesion of LS174T CD44-, but not HL60 CD44-, coated microspheres to fibrin under flow

We recently reported that CD44 is the primary fibrin, but not fibrinogen, receptor on LS174T colon carcinoma cells [8]. In view of observations suggesting that chondroitin/dermatan sulfate glycosaminoglycans on CD44 are important fibrin binding determinants [8] and that PDGF induces the expression of chondroitin/dermatan sulfate chains on CD44s in human dermal fibroblasts [11], we sought to evaluate the effect of PDGF on CD44-fibrin binding under flow. To this end, cells were cultured in the presence or absence of exogenous PDGF (100 ng/ml); a cell-free flow-based adhesion assay was employed to compare the adhesion of microspheres coated with CD44 immunopurified from either colon carcinoma cells (LS174T) which primarily express CD44v [17], [18], or human myeloid cells (HL60) which only express CD44s [17] to immobilized fibrin under flow. The use of a cell-free flow assay eliminates the potential contribution of other cell surface adhesion molecules to fibrin recognition. Of note, incubation of LS174T colon carcinoma cells with PDGF (100 ng/ml) did not affect the surface expression levels of CD44, as assessed by flow cytometry (data not shown).

**Figure 1:**
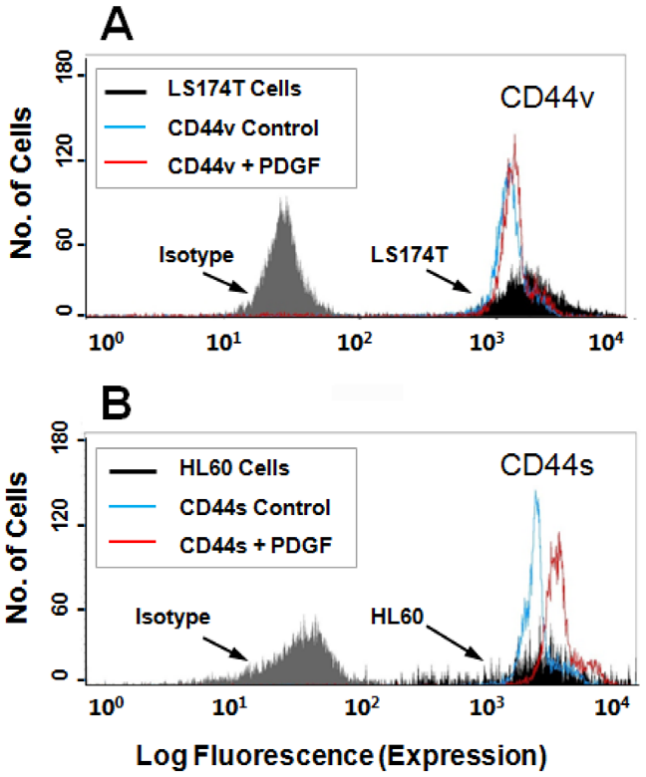
Quantification of the relative fluorescence intensity of CD44 on cells and protein-coated microspheres by flow cytometry. LS174T colon carcinoma cells or HL60 human myeloid cells were treated with either PDGF (100 ng/ml) or PBS diluent for 48–72 h. LS174T CD44 (A) or HL60 CD44s (B) was immunoprecipitated using the anti-CD44 mAbs 2C5 and 515, respectively, and adsorbed on polystyrene microspheres. CD44 expression on cells or microspheres coated with immunopurified CD44 from the corresponding cell type was quantified by single-color immunofluorescence and flow cytometry using the PE-conjugated anti-CD44 mAb 515. Background levels were determined by incubating cell or microsphere suspensions with the properly matched PE-conjugated mouse IgG isotype control antibody. Flow cytometry histograms are representative of >4 independent experiments. A minimum of 3000 events (i.e., CD44-coated microspheres or CD44-expressing cells) was analyzed on a given experiment.

**Figure 2:**
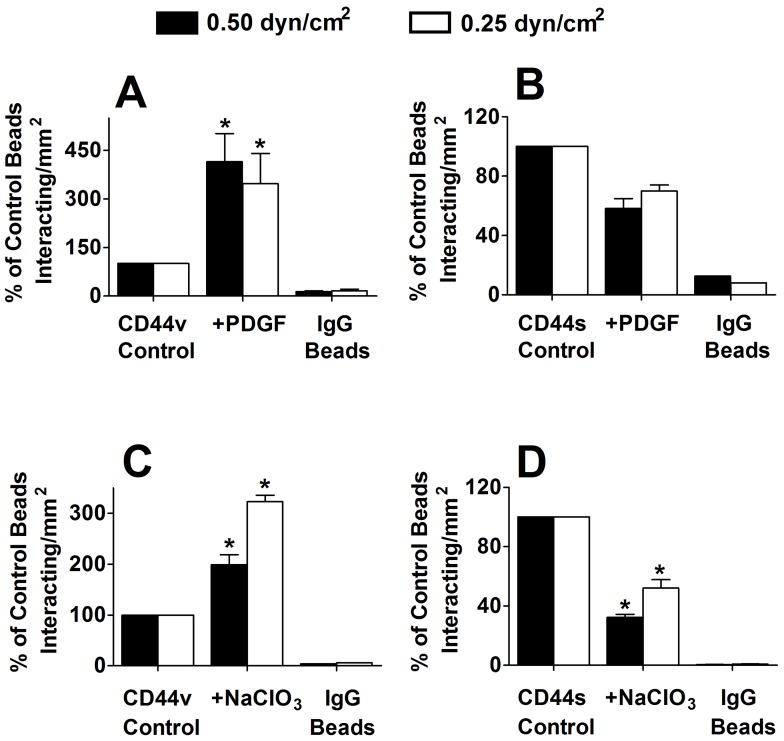
Effect of PDGF on adhesion of LS174T CD44- and HL60 CD44s- coated microspheres to immobilized fibrin in shear flow. LS174T CD44 (A) and HL60 CD44s (B) were immunoprecipitated from whole cell lysates after cell treatment for 48 h with either PDGF (100 ng/ml) or DPBS (diluent control), and adsorbed on microspheres. In other experiments, LS174T CD44 (C) and HL60 CD44s (D) were immunopurified after cell culture for 48 h in the presence and absence of sodium chlorate, and adsorbed onto beads. The CD44-coated microspheres (2×10^6^/ml) were perfused over immobilized fibrin for 5 min at the specified wall shear stresses. The numbers of interacting microspheres were quantified using phase-contrast videomicroscopy. Data are reported as percent of control CD44-bearing beads that interacted with immobilized fibrin, and represent the mean ±SEM of n = 3–4 experiments. * P<0.05 with respect to control CD44-coated microspheres. IgG-coated beads were used to assess non-specific binding to immobilized fibrin under flow.

The extent of CD44 coating on microspheres was quantified by flow cytometry, and was directly compared to the CD44 expression on the surface of intact cells. Our analysis reveals that the site density of CD44 on the microspheres was roughly equivalent to that on intact LS174T (Fig. 1A) and HL60 (Fig. 1B) cells. CD44-coated microspheres generated from PDGF-treated LS174T relative to untreated control cells displayed a markedly increased capacity to bind to immobilized fibrin under shear (Fig. 2A) despite the equivalent levels of CD44 coating as assessed by flow cytometry (geometric mean fluorescence intensity ± S.E.: 1637±188 versus 1504±205 for treated versus untreated specimens, respectively). In contrast, treatment of HL60 cells with PDGF had a modest negative effect on the ability of CD44s to interact with immobilized fibrin under flow (Fig. 2B). No significant difference was observed in the extent of LS174T CD44 or HL60 CD44s binding to fibrin substrates when the shear stress was increased from 0.25 to 0.5 dyn/cm^2^ (Figs. 2A, B). The specificity of CD44-fibrin binding was confirmed through the use of non-specific IgG-coated control microspheres, which bound minimally to immobilized fibrin in all experiments (Fig. 2).

### PDGF modulates the sulfation of chondroitin and dermatan sulfate chains on CD44

The distinctly contrasting effect of PDGF on the variant (LS174T) and standard (HL60) isoforms of CD44 is strikingly similar to that detected when sodium chlorate was used to inhibit the incorporation of sulfate groups to the CD44 molecule (Figs. 2C, D). Blocking sulfation of CD44 from LS174T colon carcinoma cells resulted in a significant increase in LS174T CD44-coated microsphere binding to immobilized fibrin under flow (Fig. 2C). In contrast, sulfate-free HL60 CD44s displayed a reduced capacity to bind fibrin (Fig. 2D). The similar effects noted with both PDGF and sodium chlorate treatments prompted us to hypothesize that PDGF inhibits the sulfation of CD44 on cells.

**Figure 3:**
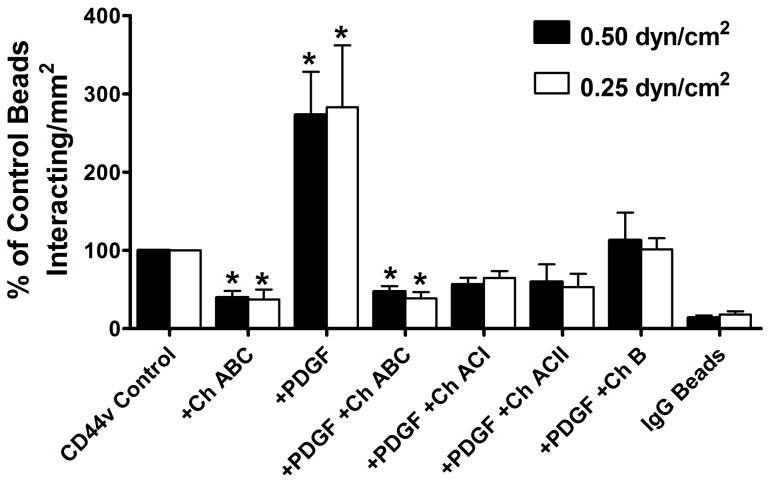
Contribution of chondroitin and dermatan sulfates to the adhesion of PDGF treated-LS174T CD44-coated microspheres to immobilized fibrin under flow. LS174T CD44-coated microspheres were generated using CD44 immunopurified from LS174T cells cultured in the presence or absence of PDGF (100 ng/ml) for 48 h. Control LS174T CD44-coated microspheres were treated with 1 U/ml chondroitinase ABC or DPBS for 1 h at 37°C. PDGF-treated LS174T CD44-coated microspheres were treated with either 1 U/ml chondroitinase ABC, B, AC II or AC I for 2 h at 37°C prior to their use in flow based adhesion assays. In all experiments, microspheres (2×10^6^/ml) were perfused over 1 mg/ml fibrin for 5 min at the specified wall shear stresses. Data are reported as percent of untreated control beads that interacted with immobilized fibrin, and represent the mean±SEM of n = 3–4 experiments. * P<0.05 with respect to untreated control microspheres. IgG-coated beads were used to assess non-specific binding to immobilized fibrin under flow.

Because a majority of the CD44 sulfation is localized on GAGs, we evaluated their contributions to LS174T CD44-fibrin interactions under flow conditions. Treatment of CD44-coated microspheres, generated from PDGF-treated LS174T or untreated control cells, with chondroitinase ABC, which degrades all forms of chondroitin and dermatan sulfates, markedly suppressed their binding to fibrin (Fig. 3), suggesting the requirement of both chondroitin and dermatan sulfate GAGs in CD44-fibrin recognition. To assess the relative contributions of the aforementioned GAGs to LS174T CD44-fibrin binding, CD44-coated microspheres were treated with chondroitinase B, which digests only dermatan sulfate [19], or chondroitinase AC II, which catalyzes the eliminative cleavage of *N*-acetylhexosaminide linkage in chondroitin sulfate [20], or chondroitinase AC I, which in addition to the AC II activity also catalyzes the efficient cleavage of the *N*-acetylgalactosaminide linkages to D-glucuronic acid in dermatan sulfate-chondroitin sulfate copolymers. Fig. 3 shows that each of these enzymatic interventions reduced the adhesion of CD44-coated microspheres prepared from PDGF-treated LS174T cells to levels at or slightly below those of microspheres generated from untreated control cells. We also determined that PDGF does not affect the expression of chondroitin sulfate chains on CD44, as assessed by flow cytometry and the use of specific mAbs to either chondroitin-4-sulfate (BE-123) or chondroitin-6-sulfate (MAB2035) or the chondroitin-4-sulfate stubs (anti-PG ΔDi-4S) exposed by chondroitinase treatment (Table 1).

**Table 1:** Expression of Chondrotin Sulfates on immunopurified CD44 from untreated and PDGF-treated LS174T cells as assessed by flow cytometry.

	Mean Fluorescent Intensity			
	anti-CD44	anti-chondroitin-4-sulfate	anti-chondroitin-6-sulfate	anti-PG ΔDi-4S
CD44v Control	1626±134	61±2.7	112±3.3	29±1.2
CD44v+PDGF	1534±147	79±1.4	99±0.3	32±0.5

CD44 expression levels on microspheres were quantified by single-color immunofluorescence and flow cytometry using the PE-conjugated anti-CD44 mAb 515. Expression of chondroitin sulfate chains on CD44-coated microspheres were quantified using the anti-chondroitin-4-sulfate (BE-123) or anti-chondroitin-6-sulfate (MAB2035) antibody or an antibody against chondroitin-4-sulfate stubs ((anti-PG ΔDi-4S) following digestion with Chondroitinase ABC) followed by incubation with a PE-conjugated anti-mouse IgG (H+L) secondary antibody. Data are reported as mean fluorescence intensity and represent the mean±SEM of n = 3 experiments.

**Figure 4:**
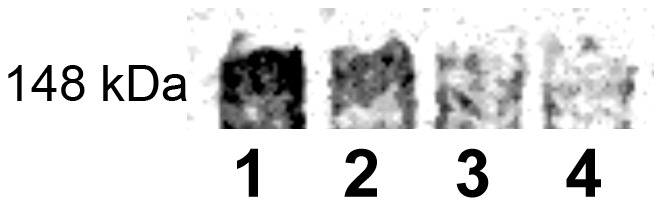
Effect of PDGF treatment on the sulfation of LS174T CD44. CD44 immunoprecipitated from untreated (control) LS174T colon carcinoma cells (lane 1) or LS174T cells treated with 100 ng/ml PDGF (lane 2) both cultured in the presence of 20 µCi/ml Na^35^SO_4_ for 48 h was subjected to SDS-PAGE. In select experiments, the untreated LS174T CD44 (lane 3) or PDGF-treated LS174T CD44 (lane 4) were incubated with 1 U/ml chondroitinase ABC for 1 h at 37°C to remove chondroitin and dermatan sulfate chains prior to being exposed to SDS-PAGE.

In view of the critical roles of both chondroitin and dermatan sulfate chains in LS174T CD44-fibrin molecular recognition and the absence of alteration of chondroitin sulfate chains on CD44 by PDGF, we hypothesized that PDGF facilitates CD44-fibrin binding via modulation of the sulfation primarily of these GAGs. To test this hypothesis, we assessed the extent of ^35^SO_4_ incorporation into the immunopurified CD44 via autoradiography by culturing cells with or without PDGF in the presence of Na^35^SO_4_. Autoradiography revealed that PDGF reduces the extent of sulfation of CD44 immunopurified from LS174T cells (Fig. 4, lane 2) as compared to the untreated control (Fig. 4, lane 1). When treated with chondroitinase ABC, the extent of CD44 sulfation is reduced in both the PDGF-treated (Fig. 4, lane 4) and respective control (Fig. 4, lane 3) samples to approximately equivalent levels. To further confirm these findings, we quantified the levels of radioactive sulfate incorporation into immunoprecipitated CD44 using liquid scintillation analysis. The level of sulfation on CD44 from PDGF-treated LS174T cells was reduced by 46.6±6% relative to that from untreated controls. Taken together, these results suggest that PDGF augments binding of LS174T CD44 to fibrin by reducing the extent of CD44 sulfation primarily on chondroitin and dermatan sulfate chains, thereby mirroring the increased LS174T CD44-fibrin binding detected with sodium chlorate treatment (Fig. 2C).

PDGF also inhibited the sulfation of HL60 CD44s by 23.5±6%, as evidenced by liquid scintillation analysis. This modest inhibition of CD44s sulfation correlates with the ∼30% reduction in the binding of HL60 CD44s-coated microspheres to immobilized fibrin under flow (Fig. 2B).

### CD44 immunopurified from PDGF-treated versus untreated LS174T cells has a higher affinity for fibrin

**Figure 5:**
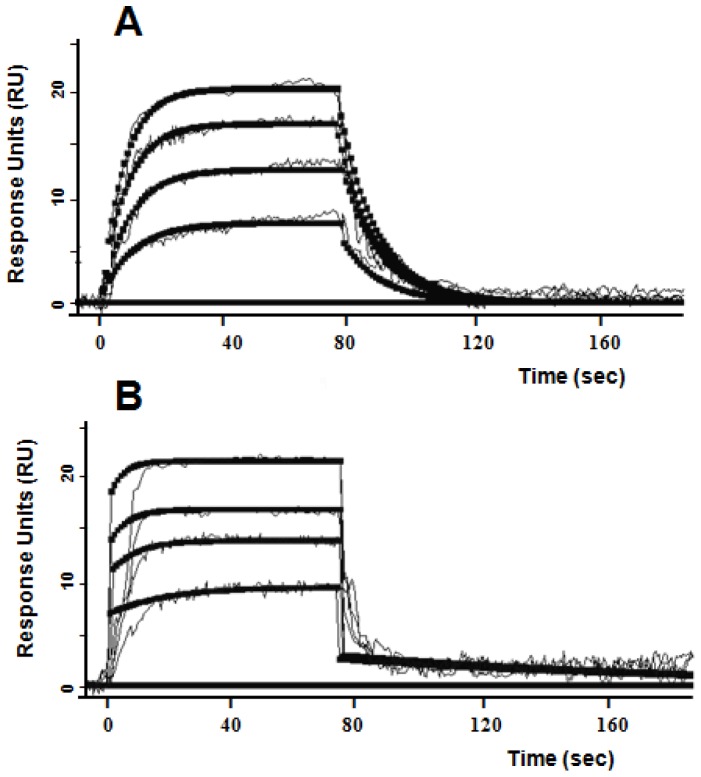
Analysis of binding of fibrin fragment β(15–66)_2_ to the immobilized CD44 from PDGF-treated and non-treated LS174T cells by surface plasmon resonance. A L1 sensor chip surface was coated with DMPC vesicles containing LS174T CD44 at 2 µL/min in 10 mM HEPES, 100 mM NaCl and 10 m M CaCl_2_. Dose-dependent binding of the Fibrin (β15–66)_2_ fragment, added at 0, 50, 120, 180, and 240 nM over immobilized CD44 from wild type LS174T cells (**A**) and to immobilized CD44 from PDGF treated-LS174T cells (**B**). The subsequent association and dissociation were monitored. Black solid squares depict the global fit of the data to a simple 1∶1 bimolecular interaction model.

To quantify the change in the affinity of LS174T CD44 for fibrin following cell treatment with PDGF, the binding constants of fibrin interaction with CD44 immunoprecipitated from PDGF-treated and untreated LS174T cells were determined by Surface Plasmon Resonance (SPR). In this assay, the immunopurified CD44 was incorporated into liposomes to mimic the physiologic orientation of the molecule on the LS174T cell membrane [21]. The soluble fibrin fragment (β15–66)_2_ was capable of binding with high affinity to immobilized CD44 immunopurified from both PDGF-treated and untreated LS174T cells (Fig. 5 and Table 2). PDGF treatment reduced by >10-fold the off rate (*k_off_*) of LS174T CD44 for fibrin (Table 2). In contrast, only a modest effect was noted on the association (*k_on_*) rate of the CD44-fibrin binding interaction (Table 2). Taken together, PGDF potentiated the affinity of CD44 for fibrin, as evidenced by a 15-fold decrease in the *K_D_* constant (Table 2).

**Table 2:** Kinetic parameters of the binding of the fibrin fragment (β15–66)_2_ to CD44v immunopurified from untreated and PDGF treated-LS174T colon carcinoma cells.

	*k*_on_ (M^−1^ s^−1^)	*k*_off_ (s^−1^)	K_D_ (M)	*χ* ^2^
CD44v control	5.46×10^5^	56.1×10^−3^	103×10^−9^	0.594
CD44v + PDGF	6.56×10^5^	4.40×10^−3^	6.71×10^−9^	0.447

The transmembrane protein CD44 immunopurified from untreated and PDGF treated-LS174T colon carcinoma cells was incorporated into a DMPC lipid bilayer and immobilized on a L1 Biacore chip. The fibrin fragment *(β15–66)_2_* was injected at different concentrations and the kinetic parameters were calculated, as previously described [8].

### PDGF treatment of LS174T CD44 increases rolling velocities over hyaluronic acid

**Figure 6:**
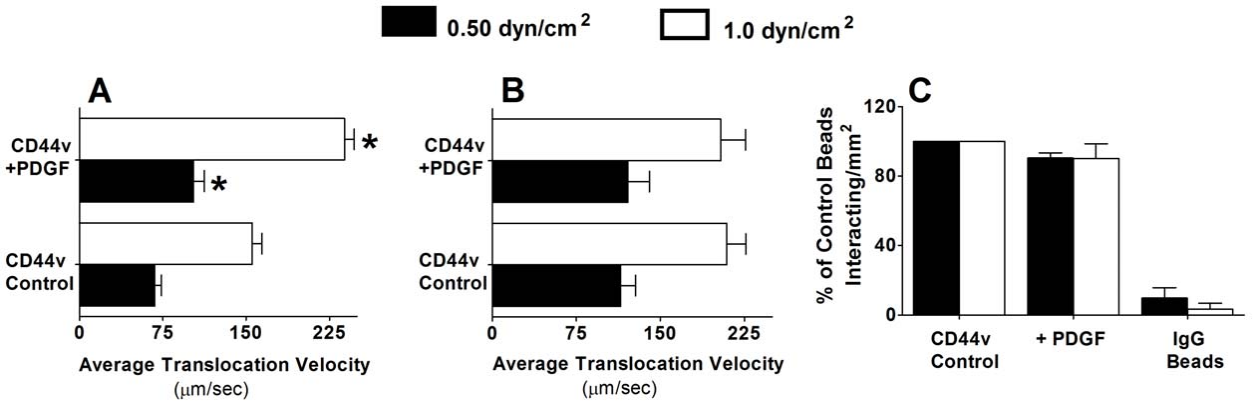
Effect of PDGF on the adhesive interactions of LS174T CD44-coated microspheres with immobilized hyaluronic acid, L-selectin or P-selectin in shear flow. CD44-coated microspheres, prepared from control and PDGF-treated LS174T colon carcinoma cells, were perfused over hyaluronan (50 µg/ml) (A) or L-selectin (1.5 µg/ml) (B) or P-selectin (1.5 µg/ml) (C) at the indicated wall shear stresses. The average translational velocity of microspheres (µm/sec) was calculated by tracking a minimum of 30 interacting microspheres (A, B). Data represent mean±SEM. * P<0.05 with respect to untreated control CD44-coated microspheres. The number of interacting CD44-coated beads with P-selectin was quantified by videomicroscopy. Data are reported as percent of untreated control CD44-bearing beads that interacted with immobilized P-selectin, and represent the mean±range of n = 2 independent experiments.

Prior work has shown that sulfation is necessary for monocytic CD44s binding to hyaluronic acid [20]. To date, there is no direct evidence that sulfation of LS174T CD44 plays a role in CD44-hyaluronic acid recognition. Thus, we examined the effect of PDGF on the adhesion of LS174T CD44 to hyaluronan. To this end, we perfused microspheres, coated with CD44 immunopurified from PDGF-treated and untreated LS174T cells, over immobilized hyaluronic acid, and quantified their adhesive interactions and rolling velocities. Although the total extent of adhesion was not altered by cell treatment with PDGF (data not shown), the rolling velocity of PDGF-treated CD44v microspheres did increase significantly relative to that of untreated controls (Fig. 6A). In contrast, PDGF failed to affect the rolling velocities of microspheres over L-selectin (Fig. 6B) or the extent of microspheres adhesion to P-selectin (Fig. 6C). These data are in agreement with our prior observation showing that selectins bind to LS174T CD44 via sialofucosylated O-linked residues presented on CD44 independent of heparan, dermatan and chondroitin sulfates [18].

## Discussion

Fibrin(ogen) plays a critical role in the metastatic dissemination of tumor cells. Microscopic observations of tumor cells, including colon carcinoma, arrested in the pulmonary vasculature reveal their intimate association with fibrin(ogen) [22]. Fibrin(ogen) clots surrounding tumor cells provide a protective shield from immunological and physiological stresses in the bloodstream, and facilitate sustained tumor cell adhesion and survival in the pulmonary vasculature [2], [3]. We recently reported that CD44 is the major functional fibrin receptor on colon carcinoma cells, and that CD44-fibrin binding interaction is dependent on chondroitin and dermatan sulfate glycosaminoglycans [8], [23]. Growth factors, including PDGF secreted from host cells such as vascular endothelium and platelets, have been reported to stimulate tumor cell growth [16], and have been implicated in tumor cell invasion and in angiogenesis [24]. Prior work has shown that PDGF enhances the glycanation of chondroitin and dermatan sulfate chains on CD44s expressed by fibroblasts and potentiates their migration into fibronectin/fibrin gels [11]. In view of this evidence, we employed an integrated approach involving cell-free flow-based adhesion assays coupled to enzymatic inhibition studies and SPR to delineate the effect of PDGF on the binding of CD44 to its major counter-receptors fibrin, selectins, and hyaluronic acid.

By immunopurifying CD44 from PDGF-treated and untreated LS174T colon carcinoma cells, which express predominantly CD44v, and HL60 myeloid cells, which exclusively express CD44s, we demonstrated the divergent effects of PGDF on CD44v versus CD44s binding to fibrin. Specifically, PDGF attenuates the binding of CD44s-coated beads to fibrin under physiologically relevant flow conditions. In contrast, PDGF enhances the adhesion of CD44v-coated beads to immobilized fibrin as assessed in cell-free flow-based adhesion assays. The similar levels of residual CD44v-fibrin adhesive interactions following enzymatic removal of chondroitin and dermatan sulfates from CD44v that is immunopurified from either untreated or PDGF-treated LS174T cells suggest that PDGF affects CD44v-fibrin recognition predominantly through modifications to these specific glycosaminoglycans. The ability of PDGF to potentiate CD44v-fibrin adhesion, while inhibiting CD44s-fibrin interactions is strikingly similar to the effects of sulfation inhibition on CD44v versus CD44s binding to fibrin [8]. We hypothesize that the removal of negatively charged sulfate groups from CD44v facilitates the binding of non-sulfated or undersulfated portions of chondroitin sulfate and dermatan sulfate glycosaminoglycans to negatively charged regions of the fibrin molecule, thereby resulting in stronger binding. Indeed, SPR measurements reveal that PDGF markedly reduces the off rate (*k_off_*) of CD44v for fibrin, while modestly increasing the association (*k_on_*) rate.

Because sulfation of the CD44 glycoprotein modulates its binding to hyaluronic acid [9], we evaluated the effect of PDGF treatment on CD44v-hyaluronan adhesive interactions using a cell-free flow-based adhesion assay. Our analysis reveals that PDGF significantly increases the rolling velocities of CD44v-coated microspheres over immobilized hyaluronic acid under flow without affecting the extent of adhesion. Together, these data suggest that the reduction in the sulfation of glycosaminoglycans on CD44v induced by PDGF may decrease the off rate without altering the association rate of CD44v-hyaluronan binding.

In summary, this study offers a novel perspective into the mechanism by which PGDF regulates CD44-dependent binding of metastatic colon carcinoma cells to fibrin(ogen) pertinent to the process of cancer metastasis.

## Materials and Methods

### Reagents and monoclonal antibodies

Fibrinogen (von Willebrand factor-, plasminogen- and fibronectin-free) and plasmin were from Enzyme Research Laboratories (South Bend, IN). Thrombin, human IgG, chondroitinase ABC (*Proteus vulgaris*), chondroitinase B (*Flavobacterium heparinum*), chondroitinase AC II (*Arthrobacter aurescens*), PDGF, hyaluronic acid, sodium chlorate, and polycarbonate filter membranes with a pore size of 100 nm were purchased from Sigma-Aldrich (St. Louis, MO). The chondroitinase AC I (*Flavobacterium heparinum*) and the antibody to chondroitin-4-sulfate stubs (anti-PG ΔDi-4S) were from Seikagaku Corporation (Tokyo, Japan). The anti-chondroitin-4-sulfate (BE-123) and anti-chondroitin-6-sulfate (MAB2035) were obtained from Chemicon (Billerica, MA). The anti-human CD44 mAb, 2C5, P-selectin-Fc chimera and L-selectin-Fc chimera were obtained from R & D systems (Minneapolis, MN). The unlabeled and PE-conjugated mouse anti-human CD44 (515), and PE-labeled mouse IgG1 isotype control were from BD Biosciences (San Jose, CA). The 1,2-dimyristoyl-sn-glycero-phosphatidylcholine (DMPC) was obtained from Avanti Polar Lipids, Inc (Alabaster, AL). The L1 sensor chip was purchased from Biacore (GE Healthcare).

### Cell culture, whole cell lysis and immunoprecipitation of CD44

LS174T human colon adenocarcinoma cells and HL60 human myeloid cells (American Type Culture Collection; Manassas, VA) were cultured in the presence or absence of PDGF (100 ng/ml) for 48 h prior to cell lysis and CD44 immunoprecipitation. LS174T or HL60 whole cell lysates were prepared by membrane disruption using 2% Nonidet P-40 followed by differential centrifugation. CD44v was immunoprecipitated from LS174T colon carcinoma cell lysate with the anti-CD44 mAb 2C5, whereas CD44s was immunopurified from HL60 human myeloid cells with the anti-CD44 mAb 515, using recombinant protein G-agarose beads (Invitrogen) [17], [25], [26]. We have previously shown that our protocol results in immunoprecipitates that typically contain the antigen along with a very small amount of antibody released from the protein G beads, whereas 99% of the antibody remains bound to the protein G beads [8], [17].

### Cell treatments

In select experiments, LS174T or HL60 cells were cultured for 48 h in medium containing 60 or 20 mM sodium chlorate, respectively, or DPBS diluent (untreated control) prior to lysis and CD44 immunopurification. To cleave specific glycosaminoglycans (GAGs) from CD44, CD44-coated microsphere suspensions were incubated for 1 h at 37°C with either 1 U/ml *Proteus vulgaris* chondroitinase ABC (which degrades all forms of chondroitin sulfate as well as dermatan sulfate), chondroitinase B (which digests only dermatan sulfate), *Arthrobacter aurescens* chondroitinase AC II (which catalyzes the eliminative cleavage of N-acetylhexosaminide linkage in chondroitin sulfate) or *Flavobacterium heparinum* chondroitinase AC I (which in addition to the AC II activity also catalyzes the cleavage of N-acetylgalactosaminide linkages to D-glucuronic acid in dermatan sulfate-chondroitin sulfate copolymers). Site densities of CD44 adsorbed onto microspheres following enzymatic treatments were determined by flow cytometry and confirmed to be equivalent to untreated (no enzyme) controls before use in adhesion assays.

### Preparation of CD44-coated microspheres

Prior to protein binding, 10-µm polystyrene microspheres (2.5×10^7^ microspheres/ml; Bangs Labs, Fishers, IN) were washed 3X with DPBS, followed by 2X with citrate-phosphate buffer (pH 3.0). After a 1 h incubation at room temperature (RT), the microspheres were washed once more with the citrate-phosphate buffer, 3X with DPBS and 2X with binding buffer (0.2 M carbonate/bicarbonate buffer, pH 9.2). Immunoprecipitated CD44 from LS174T or HL60 whole cell lysate or human IgG was diluted to desired concentrations with binding buffer, and incubated with the microspheres overnight at 4°C with constant rotation. Microspheres were washed 2X with DPBS and subsequently blocked with DPBS, 1% BSA for 1 h at RT. Subsequently, microspheres were resuspended (2×10^6^ microspheres/ml) in DPBS, 0.1% BSA for use in flow cytometric and flow chamber assays.

### Flow cytometry

CD44 expression levels on LS174T and HL60 cells and CD44-coated microspheres were quantified by single-color immunofluorescence and flow cytometry (FACSCalibur; BD Biosciences) using the PE-conjugated anti-CD44 mAb 515. Background levels were determined by incubating cell or microsphere suspensions with the properly matched PE-conjugated mouse IgG isotype control antibody. Expression of chondroitin sulfate chains on CD44-coated microspheres was quantified using the anti-chondroitin-4-sulfate (BE-123) or anti-chondroitin-6-sulfate (MAB2035) followed by incubation with a PE-conjugated anti-mouse IgG (H+L) secondary antibody (Vector Laboratories, Burlingame, CA). In select experiments, chondroitinase ABC-treated CD44-coated microspheres were incubated with the antibody to chondroitin-4-sulfate stubs (anti-PG ΔDi-4S) followed by incubation with a PE-labeled secondary antibody.

### Flow-based adhesion assays

Fibrinogen-coated surfaces were prepared by incubating 1 mg/ml vWf-, plasminogen-, and fibronectin-free fibrinogen in DPBS on untreated 35-mm polystyrene suspension culture dishes overnight at 4°C. Fibrin-coated surfaces were prepared by washing the immobilized fibrinogen 3X with DPBS, and subsequently incubating with 2 U/ml thrombin for 2 h at 37°C. Hyaluronic acid-coated surfaces were generated by incubating 50 µg/ml hyaluronic acid in binding buffer on untreated polystyrene dishes overnight at 4°C. Selectin-coated surfaces were prepared by coating dishes with anti-human IgG Fc overnight, washing with DPBS, followed by a 2 h incubation with 1.5 µg/ml P- or L-selectin-Fc chimera [18], [26]. Plates were then washed with DPBS and blocked with 1% BSA for 1 h prior to their use in flow-based adhesion assays. Suspensions of microspheres (2×10^6^/ml) were perfused over fibrin- or hyaluronic acid- or selectin-coated dishes using a parallel plate flow chamber (250 µm channel depth, 5.0 mm channel width) for 5 min at 37°C. The number of interacting microspheres was then quantified by phase-contrast videomicroscopy, as previously described.

### Quantification of CD44 sulfation

To determine the degree of sulfation of CD44, LS174T or HL60 cells were treated with 20 µCi/ml or 10 µCi/ml of Na^35^SO_4_ (Perkin-Elmer, Waltham, MA) for 48 h in the presence or absence of PDGF (100 ng/ml). Immunoprecipitated CD44 was then separated via SDS-PAGE on a 4–12% Bis-Tris NuPAGE gel (Invitrogen), fixed in 10% acetic acid/50% methanol for 30 min, incubated in Amplify fluorographic reagent (GE Healthcare, Buckinghamshire, UK) for 30 min, and dried onto paper before exposure and scanning using a Typhoon 9410 Variable Mode Imager (GE Healthcare). To determine the level of radioactive sulfates (^35^SO_4_) incorporated into CD44, 50 µl of immunoprecipitated protein solutions (of prescribed concentrations quantified with the NanoDrop 2000 spectrophotometer using A280 absorbance readings) were diluted with 10 ml of scintillation fluid, and the counts per minute were quantified on a Packard Tri-Carb Liquid Scintillation Counter.

### Binding studies by SPR assay

The interaction of the fibrin fragment β(15–66)_2_ with the immobilized CD44, immunopurified from PDGF-treated and non-treated LS174T colon carcinoma cells, was studied by SPR using the BIAcore 3000 biosensor (BIAcore AB, Uppsala, Sweden), which measures the association/dissociation of proteins in real time. Immobilization of the CD44 glycoprotein to the SPR sensor chip was performed using the BIAcore L1 chip. In brief, on two channels of the L1 sensor chip surface, DMPC proteoliposomes containing CD44 from PDGF-treated and non-treated LS174T cells were immobilized. Pure liposomes were immobilized on a third channel as a reference control. The fibrin fragment β(15–66)_2_ was diluted in the running buffer (0.2 µm filtered, degassed HBS), and 60 µl injections were done at prescribed concentrations with a flow rate of 40 µl/min. The chip surface was regenerated using 50 µl of NaOH (20 mM). Experimental data were performed in triplicate and analyzed using BIAevaluation 4.1 software. Kinetic constants for association (*k_on_*) and dissociation (*k_off_*) were estimated by global analysis of the association/dissociation curves using the 1∶1 Langmurian interaction model, and the dissociation equilibrium constant (*K_d_*) was calculated as *K_d_* = *k_off_*/*k_on_*.

### Statistics

Data are expressed as the mean±SEM, unless otherwise stated. Statistical significance of differences between means was determined by ANOVA. If means were shown to be significantly different, multiple comparisons by pairs were performed by the Tukey test. Probability values of p<0.05 was selected to be statistically significant.
